# Primary marginal zone B-cell lymphoma of the cavernous sinus: a case report and review of the literature

**DOI:** 10.1186/s12880-021-00556-w

**Published:** 2021-02-12

**Authors:** Cheng-Chun Yang, Tai-Yuan Chen, Yu-Kun Tsui, Ching-Chung Ko

**Affiliations:** 1 Department of Medical Imaging, Chi Mei Medical Center, No. 901, Zhonghua Rd., Yongkang Dist., Tainan City, 710 Taiwan, ROC; 2 Graduate Institute of Medical Sciences, Chang Jung Christian University, Tainan, Taiwan; 3 Department of Health and Nutrition, Chia Nan University of Pharmacy and Science, Tainan, Taiwan

**Keywords:** CNS lymphoma, Marginal zone B-cell lymphoma, Cavernous sinus, MRI, DWI, ADC

## Abstract

**Background:**

Primary lymphoma of the cavernous sinus is a rare form of extranodal non-Hodgkin lymphoma, of which very few cases have been reported in the published literature. This report presents the MRI findings with apparent diffusion coefficient (ADC) value in an exceedingly rare primary marginal zone B-cell lymphoma (MZBCL) of the cavernous sinus.

**Case presentation:**

The case in this study is a 59-year-old immunocompetent male patient with a 2-month history of right ptosis and blurred vision. Right third cranial nerve palsy and binocular diplopia were observed upon neurological examination. Preoperative brain CT showed an extra-axial enhancing mass lesion in the right cavernous sinus. On MRI, ipsilateral internal carotid arterial encasement was noted without causing stenosis of the vessel. Isointense signal on T1-weighted and T2-weighted images, homogeneous contrast enhancement, and diffusion restriction were also observed. The mean ADC value of the tumor is 0.64 × 10^–3^ mm^2^/s (b value = 1000 s/mm^2^). Subtotal resection of the tumor was performed, and improvement of clinical symptoms were observed. The pathologic diagnosis of MZBCL was established by immunohistochemical examinations.

**Conclusions:**

Primary MZBCL of the cavernous sinus is exceedingly rare, and preoperative confirmation poses a major challenge with CT and conventional MRI only. In this case, preoperative quantitative ADC value is shown to offer valuable additional information in the diagnostic process.

## Background

Primary central nervous system lymphoma (PCNSL) is a rare form of extranodal non-Hodgkin lymphoma (NHL) typically limited to the neuroaxis without systemic involvement. It represents approximately 1% of NHLs and 4% of all intracranial neoplasms [1, 2]. The brain parenchyma is the most common site of involvement, followed by the eye, the leptomeninges, and the spinal cord [3]. Most PCNSLs are aggressive lymphomas that typically affects immunocompromised patients and carry a dismal prognosis [3, 4]. Diffuse large B-cell lymphoma (DLBCL) is the most common histologic type, accounting for 90% of the cases [5]. The PCNSL usually presents as tumor mass located in the periventricular region of the brain parenchyma and exhibits pronounced contrast enhancement. Tumor necrosis with heterogeneous enhancement are more prevalent in the setting of immunocompromise [6]. However, PCNSL arising from the cavernous sinus is an extremely rare condition. The radiologic features of primary cavernous sinus lymphoma remain unclear owing to its rarity. This report details comprehensive preoperative CT and MRI findings with apparent diffusion coefficient (ADC) value in a case of primary marginal zone B-cell lymphoma (MZBCL) arising from the cavernous sinus. Published works pertaining to primary cavernous sinus lymphoma are also reviewed.

## Case presentation

**Fig. 1:**
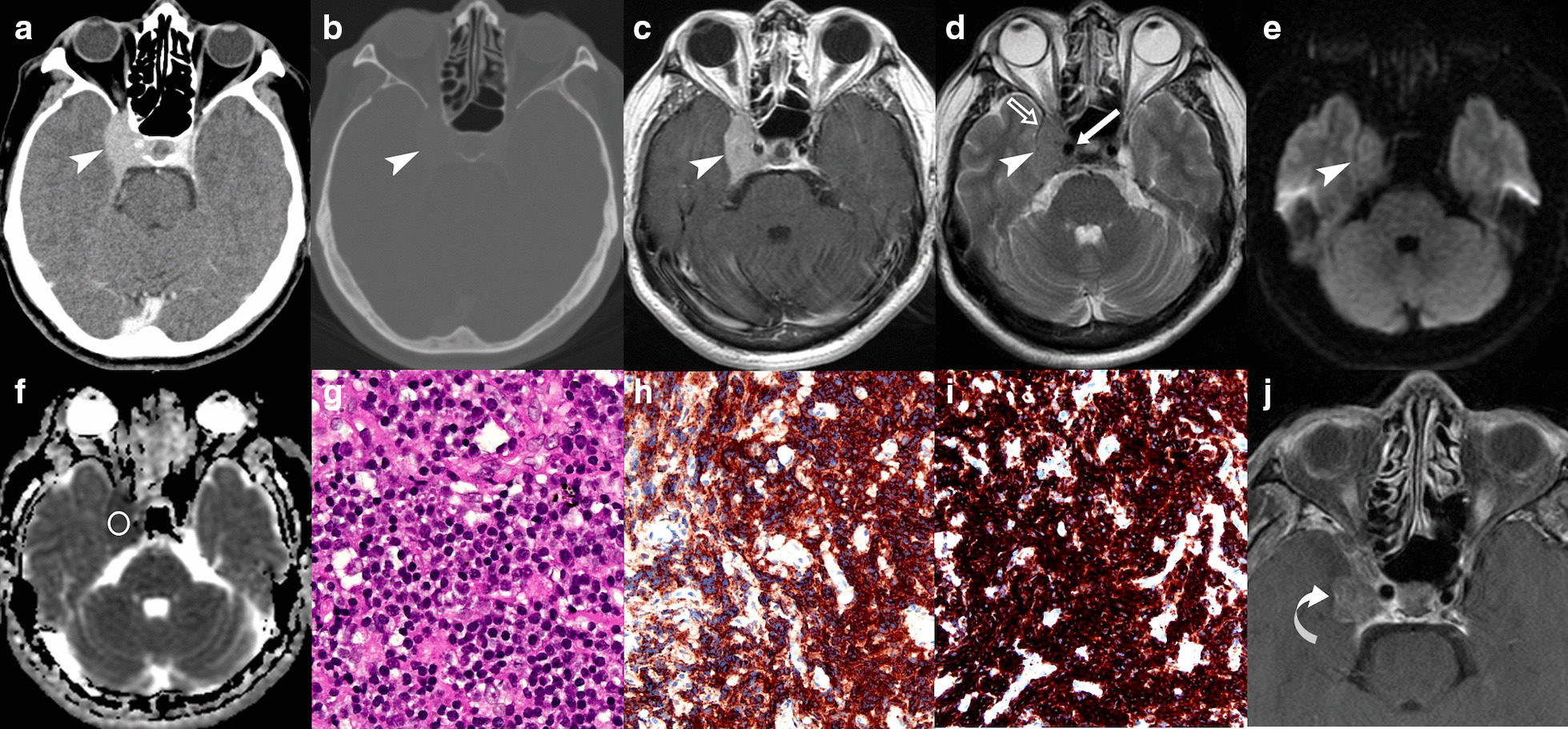
A 59-year-old man with pathologically confirmed primary MZBCL of the right cavernous sinus. **a** Axial contrast-enhanced (CE) brain CT shows a bulging mass lesion (arrowhead) in the right cavernous sinus and measures 2.0 × 3.3 cm in size. **b** Axial bone window CT image revealed no surrounding skull bone destruction or hyperostosis around the tumor (arrowhead). **c** Axial CE T1WI shows homogeneous contrast enhancement of the tumor (arrowhead) after gadolinium administration. **d** The extra-axial origin of the tumor (arrowhead) is identified based on indentation of the right temporal dura (open arrow) on axial T2WI. In addition, encasement of ipsilateral cavernous internal carotid artery is observed without causing narrowing of the vascular lumen (white arrow). **e** Diffusion-weighted image (DWI) shows isointensity in the tumor (arrowhead). **f** The measured apparent diffusion coefficient (ADC) value (circular region of interest) is 0.64 × 10^–3^ mm^2^/s (b value = 1000 s/mm^2^), indicating diffusion restriction as compared with brain white matter. **g** The resected tumor is composed of a diffuse infiltration of small to median-sized lymphocytes mixed with plasma cells (**h**, **e**, × 200). **h**, **i** Positive staining for pan B cell markers including CD20 (**h**) and CD79a (**i**) on immunohistochemical examination (original magnification × 400) are observed. **j** A stable residual mass with contrast enhancement (curved arrow) is observed on axial CE T1WI 2 years after surgical resection

The case in this study is a 59-year-old male patient with unremarkable medical history and a 2-month duration of right ptosis and blurred vision. No headache, fever, weight loss or nocturnal sweating is documented. Neurological examination revealed palsy of the right third cranial nerve (CN) and binocular diplopia. Pupillary light reflex and corneal reflex were intact. Routine laboratory data including complete blood count and urinalysis are within normal limits. Brain CT revealed an enhancing mass lesion in the right cavernous sinus (Fig. 1a) without surrounding skull bone destruction or hyperostosis (Fig. 1b). On brain MRI, an extra-axial tumor exhibiting isointense signals on T1-weighted and T2-weighted images and homogeneous contrast enhancement is observed (Fig. 1c, d). In addition, diffusion restriction is observed on diffusion-weighted imaging (DWI) with a low ADC value of 0.64 × 10^–3^ mm^2^/s (b value = 1000 s/mm^2^) (Fig. 1e, f). The initial diagnoses made by the reporting neuroradiologist included meningioma, neurogenic tumor, and PCNSL. The patient underwent subtotal resection of the tumor via a right extended pterion approach. Intraoperatively, a soft and white tumor adhering to the wall of the cavernous sinus was identified, which was then meticulously resected without causing injury to the internal carotid artery (ICA). Histological examination revealed diffuse infiltration of the submitted specimen by small- to medium-sized lymphoid cells mixed with plasma cells (Fig. 1g). The final histopathologic diagnosis is MZBCL by immunohistochemical studies (Fig. 1h, i). A generalized systemic survey consisted of whole-body CT, positron emission tomography (PET) scan, and bone marrow biopsy. The results revealed no evidence of extracranial involvement. Clinical stage I_E_ (extranodal disease limited to a single site) was thus designated according to the 2014 Lugano classification [7]. After surgery, the patient experienced relief of right ptosis and blurred vision. Adjuvant radiotherapy and chemotherapy were also implemented for residual tumor. Residual tumor and post-irradiation changes remained stable on follow-up MRI studies for more than 2 years (Fig. 1j).

## Discussion and conclusions

PCNSL arising from the cavernous sinus is a rare occurrence with only 21 known cases published in available literature in the English language. Amongst these cases, 8 are histologically confirmed MZBCL (Table 1). However, there have so far been no documented studies on preoperative comprehensive MRIs and ADC values in primary MZBCL of the cavernous sinus. The cavernous sinus can be involved by a myriad of diseases including neoplasms, vascular lesions, and infectious or inflammatory processes. In regards to neoplasms, the cavernous sinus is rarely involved by lymphoma, either primary or metastatic. Reported clinical symptoms of cavernous sinus lymphoma include visual disturbance, headache, ophthalmoplegia, cavernous sinus syndrome, or clinical features resembling trigeminal neuralgia and Tolosa–Hunt syndrome (Table 1).

**Table 1:** Primary central nervous system lymphoma of the cavernous sinus in published studies

References	Age/sex	Immune status	Histologic type	Symptoms	Involvement site	Treatment	CT/MRI	DWI/ADC	Out-come
de la Fuente et al. [8]	30 F	HIV (–)	MZBCL	Facial pain	CS	Surgery, R/T	−/−	N/A	CR
	51 F	HIV (–)	MZBCL	Focal paresthesia and numbness	Left CS	C/T, R/T	−/−	N/A	CR
	48 F	HIV (–)	MZBCL	Cranial nerve palsy	Bilateral CS	C/T	−/−	N/A	CR
	50 F	HIV (–)	MZBCL	Cranial nerve palsy	CS	Surgery, R/T	−/−	N/A	CR
Suresh et al. [9]	24 F	N/A	MZBCL	Right painless vision loss, bitemporal visual field defect	Left CS, falx, tentorium	Surgery, C/T	−/+	N/A	N/A
Dultra et al. [10]	63 M	HIV (–)	NHK B cell lymphoma	Frontal headache, right facial pain, diplopia	Bilateral CS, sella	Surgery, R/T	−/+	N/A	CR
Ko et al. [11]	70 F	N/A	DLBCL	Facial pain, complete right ptosis, diplopia	Right CS, right orbit	C/T	−/+	N/A	CR
Demirkaya et al. [12]	4 M	N/A	NHK	Right ptosis, pupil dilatation, ophthalmoplegia	Right CS	Surgery, C/T	−/+	N/A	CR
Sadruddin et al. [13]	17 F	N/A	TLBL	Rapidly progressive right headache, diplopia, facial numbness	Right CS	Surgery, C/T, R/T	−/+	N/A	CR
Famoso et al. [14]	46 F	Immuno-competent	MZBCL	Right exophthalmos, ptosis, retro-orbital pain	Right CS, sella, orbital meninges	Surgery, rituximab	−/−	N/A	PR
Razaq et al. [15]	61 F	HIV (–)	MZBCL	Headache, CN III palsy	Left CS, left optic nerve	R/T, rituximab	−/−	N/A	CR
Choi et al. [16]	12 M	N/A	DLBCL	Visual disturbance, periorbital pain	Bilateral CS, dura, sphenoid bone	C/T	+/+	N/A	CR
Nakamura et al. [17]	69 M	N/A	*highly malignant B cell lymphoma	Diplopia, left facial numbness, dysarthria, dysphagia (Garcin syndrome)	Left CS, occipital and clival bone	N/A	−/+	N/A	N/A
Ronson et al. [18]	53 F	N/A	DLBCL	Diplopia, headache, left mouth paresthesia	Right CS, right sphenoid sinus extension	Surgery, C/T, R/T	−/−	N/A	N/A
Sanjeevi et al. [19]	46 F	HIV (–)	MZBCL	Left side headache, ophthalmalgia, decreased visual acuity	Left CS, left orbital apex	Surgery, R/T	−/+	N/A	CR
Arimoto et al. [20]	59 F	HIV (–)	*diffuse small B cell lymphoma	Diplopia on right lateral gaze, right facial hypesthesia	Right CS	Surgery, R/T	−/+	N/A	CR
Jaiswal et al. [21]	40 M	HIV (–)	DLBCL	Right sided headache, tinnitus, hearing impairment, ataxia	Right CPA, right CS, right orbit	Surgery, C/T, R/T	+/+	N/A	CR
Roman-Goldstein et al. [22]	37 M	Immuno-competent	N/A	Retro-orbital headache, diplopia	Right CS	C/T	−/+	N/A	CR
	62 M	Immuno-competent	N/A	Headache, diplopia	Left CS	Surgery, C/T, R/T	−/−	N/A	Died at 18 month
	51 F	Immuno-competent	N/A	Diplopia	Left CS	Surgery, C/T	−/−	N/A	N/A
Nakatomi et al. [23]	77 M	HIV (–)	DLBCL	Left facial hypesthesia, diplopia	Left CS, petroclival dura	Surgery, C/T, R/T	−/+	N/A	Died at 31 month

*Pathologic diagnoses not applicable to WHO 2016 classification, CPA, cerebellopontine angle; CS, cavernous sinus; CR, complete response; C/T, chemotherapy; DLBCL, diffuse large B-cell lymphoma; HIV, human immunodeficiency virus; N/A, not available; NHK, non-Hodgkin lymphoma; PR, partial response; R/T, radiotherapy; TLBL, T-cell acute lymphoblastic lymphoma

MZBCL, a low-grade non-Hodgkin lymphoma derived from the postgerminal center, is best known for constituting the mucosal associated lymphoid tissue lymphoma (MALT lymphoma or MALToma) of the gastrointestinal tract associated with chronic infection of Helicobacter pylori [24]. In addition, recent studies had identified various autoimmune, chromosomal, and genetic factors to also be implicated in the pathogenesis of MZBCL [25]. CNS involvement of extranodal MZBCL is uncommon, and when it occurs, the most commonly involved structure is the dura (80% of the cases), followed by the brain parenchyma and other sites [26]. Radiologically, the disease typically presents as a dural-based mass, a feature rendering it easily mistaken for meningioma [27]. The pathogenesis of primary dural MZBCL is unclear, since the dura is devoid of organized lymphoid tissue. Some authors hypothesized that MZBCL may arise from meningothelial cells, which are tasked with the immunological protection of the brain from bacterial infection [28, 29]. Likewise, the cavernous sinus also contains dura matter and can, at least theoretically, be involved by MZBCL, as is the case in our report. On the other hand, MZBCL is the most common histologic subtype (up to 75%) in ocular adnexal lymphoma [30].

Common differential diagnoses of cavernous tumors include meningioma, schwannoma, metastasis, Tolosa–Hunt syndrome, and hemangioma. From a radiological point of view, differentiation amongst these lesions can be challenging with conventional MRI only, since all may exhibit similar signal intensities and enhancement patterns. However, ADC values may offer additional diagnostic value in differentiating these lesions. In PCNSLs, diffusion restriction with low ADC values between 0.63 × 10^–3^ and 0.71 × 10^–3^ mm^2^/s have been reported [10, 31, 32]. De la Fuente et al. [8] have reported the median ADC value of 0.598 × 10^–3^ mm^2^/s in six dural MZBCL cases. Although the final diagnosis of MZBCL of the cavernous sinus needs to be confirmed by immunohistochemistry, the preoperative quantitative low ADC value (0.64 × 10^–3^ mm^2^/s) in our case offers valuable additional information in the diagnostic process. On conventional MRI, ICA encasement with vascular stenosis is characteristically present in the case of cavernous sinus meningiomas [33]. In contrast, patent ICA vascular lumen without narrowing is observed in our case. Most meningiomas are benign tumors and typically do not exhibit diffusion restriction on DWI [34]. Although rare high-grade (atypical and malignant) meningiomas may sometimes demonstrate diffusion restriction, higher ADC values of 0.78 × 10^–3^ mm^2^/s to 0.8 × 10^–3^ mm^2^/s had been reported in these high-grade meningiomas as compared with our case [35]. Furthermore, occurrence of high-grade meningiomas arising from the cavernous sinus has never been reported. Similarly, most of the nerve sheath tumors arising from the traversing cranial nerves or sympathetic plexus are benign neoplasms that do not exhibit diffusion restriction [36]. Metastases to the cavernous sinus can be more readily recognized based on a known clinical history of cancer, which may occur via direct invasion, perineural spread from the adjacent nasopharynx and bone, or hematogenous spread. Head and neck cancers are the most commonly identified culprits; rare instances of distant metastases from lung, breast and prostate malignancies have also been reported [37]. Tolosa–Hunt syndrome is essentially a clinical diagnosis of exclusion. It describes an idiopathic inflammatory process characterized by painful ophthalmoplegia secondary to inflammation surrounding the cavernous sinus. The usual presentation is that of an enhancing soft tissue lesion involving both the orbital apex and cavernous sinus, features different from those observed in our case [38]. Hemangiomas are rare vascular lesions involving the cavernous sinus which, similar to those occurring elsewhere, exhibit marked hyperintensity on T2WI and progressive contrast filling (centripetal enhancement) on dynamic contrast-enhanced T1WI [39]. In our case, theses differential diagnoses can be excluded in sequence by clinical history and MRI findings. Because MZBCL is extremely radiosensitive, regional treatment consisting of focal resection followed by radiotherapy has been shown to achieve complete response in most cases of dural MZBCL [3, 5]. Therefore, preoperative recognition of this rare entity makes it possible to avoid unnecessarily aggressive surgical intervention.

To the best of our knowledge, this report is the first to mention and discuss preoperative ADC values of the primary cavernous sinus MZBCL. This case suggests that the combination of preoperative MRI and ADC values may offer helpful additional information regarding to the diagnosis and treatment planning for lymphoma of the cavernous sinus.

## Data Availability

All data generated or analyzed in this study are included in this published article.

## Abbreviations

ADC: Apparent diffusion coefficient
DLBCL: Diffuse large B-cell lymphoma
DWI: Diffusion-weighted image
MZBCL: Marginal zone B-cell lymphoma
PCNSL: Primary CNS lymphoma
PET: Positron emission tomography
T1WI: T1-weighted image
T2WI: T2-weighted image
